# Extraction, purification, and structure characterization of polysaccharides from *Crassostrea rivularis*

**DOI:** 10.1002/fsn3.695

**Published:** 2018-07-20

**Authors:** Xiaoming Qin, Xiuping Fan, Lianyi Zhang, Huina Zheng, Chaohua Zhang, Jianjun Yuan

**Affiliations:** ^1^ South China Sea Bio‐Resource Exploitation and Utilization Collaborative Innovation Center Zhanjiang China; ^2^ Guangdong Provincial Key Laboratory of Aquatic Product Processing and Safety Zhanjiang China; ^3^ National Research and Development Branch Center for Shellfish Processing Zhanjiang China; ^4^ College of Food Science and Technology Guangdong Ocean University Zhanjiang China; ^5^ Fujian Province Key Laboratory for the Development of Bioactive Material from Marine Algae Quanzhou China

**Keywords:** chemical characterization, *Crassostrea rivularis*, glycogen, polysaccharide

## Abstract

Crude polysaccharide was prepared from *Crassostrea rivularis* by 30% (w/v) potassium hydroxide solution at 90°C for 120 min. Three fractions (OG1, OG2, and OG3) were purified by DEAE‐52 cellulose and Sepharose 2B gel column chromatography. The chemical structures were determined using gas chromatography (GC), high‐performance gel permeation chromatography (HPGPC), Fourier‐transform infrared (FT‐IR) spectroscopy, and ^1^H and ^13^C nuclear magnetic resonance (NMR) spectroscopy. The results indicated that OG1 was composed of rhamnose and little mannose (8.71%), the ratio of Rha: Gal: Xyl: Fuc in OG3 were 14:5.5:3:1. And their average molecular weights (Mw) were about 1.66 × 10^6^ and 2.33 × 10^6^ Da, respectively. OG2 was composed only of glucose (98.23%), which means it was glycogen. OG2 was consisted mainly of →4)‐ α‐D‐Glc‐(1→, with the branch chain every 6.5 glucose residues on average, which is →4,6)‐α‐D‐Glc‐(1→ and trace amount of α‐D‐Glc‐(1→ branched units. The Mw was 2.27 × 10^6^ Da. It provides the bases for the bioactivity research.

## 1 INTRODUCTION

Mollusks are an excellent source of high‐quality nutrition in China as well as other parts of the world and have been used as food products and traditional Chinese medicinal drugs for centuries. In recent years, the water‐soluble polysaccharides present in mollusks have become the focus of intense interest due to their various bioactivities, such as immunomodulation activity (Dai, Zhang, Zhang, & Wang, 2009; Ovodova et al., 1992), antioxidant, and hepato‐protective activities (Jiang, Wang, Liu, Gan, & Zeng, 2011; Liao et al., 2015).

Oysters are rich in polysaccharides. The pacific Oyster (*Crassostrea gigas*)‐derived polysaccharides (OPS) have been shown to modulate the T helper Th1/Th2 immunobalance toward the Th1‐dominant direction in antigen‐primed splenocytes (Cheng, Wu, Wu, & Jan, 2016). Yang et al. (2013) also found the oyster glycogen has the lymphocyte proliferation effect. Functional studies have been conducted on oyster polysaccharides (OPS), including their inhibitory effects on influenza virus reproduction (Li, Hou, & Lai, 2009), immunostimulatory activity (Li, Wang, Zhang, Wu, & Liu, 2013), antimicrobial activity (Tian et al., 2013), as well as the complement activity of oyster glycogens (Song, Zhu, Yang, & Sun, 2011) and their hepato‐protective effects (Hu, Wu, Fan, & Liu, 2014; Shi et al., 2015). But the structure of polysaccharide and the structure–activity relationship have still not researched by people.

*Crassostrea rivularis* lives in the conjunction regions of rivers and the sea. It is the main economical mollusk along the South China Sea and often aquacultured in Guangdong, Guangxi and Hainan provinces. It is desired for its flavor, protein, low fat, and high glycogen content. Qin, Lin, Zhang, and Zhang (2011) researched the sobering effects of the components in oyster meat and found that polysaccharide and taurine were the major sobering substances in oyster meat. In order to research the sobering‐up and antifatigue functions of polysaccharide from *C. rivularis,* we isolated the polysaccharide and analyzed the chemical structure in this study. Three isolation methods were compared in the polysaccharide extraction; then, the alkaline method was selected to extract the crude polysaccharide and purified using DEAE‐cellulose and Sepharose 2B gel chromatagraphy. The preliminary characterizations were investigated using high‐performance gel permeation chromatography (HPGPC), Fourier‐transform infrared (FT‐IR) spectroscopy, and ^1^H and ^13^C nuclear magnetic resonance (NMR) spectroscopy.

## 2 MATERIALS AND METHODS

### 2.1 Materials

*Crassostrea rivularis* were purchased from Zhanjiang Dongfeng aquatic products market (Zhanjiang City, China). They were all collected from the South Sea. The shell was removed, and the remainder was homogenized with cold distilled water (w/w, 1:1), then frozen at −70°C for isolation and analysis.

Arabinose, rhamnose, fucose, xylose, galactose, glucose, mannose, DEAE‐52 cellulose, and Sepharose 2B gel were obtained from Sigma Chemical Co. (St. Louis, MO, USA).

### 2.2 Experimental methods

The chemical composition of *C. rivularis* was assessed using the standard methods as follows: water content (GB/T 5009.3‐2010), ashes (GB/T 14772‐2008), fats (GB/T 14772‐2008), and crude protein (GB/T 5009.5‐2010). Moisture was determined by oven‐drying at 105°C to constant weight, crude fat was extracted using the Soxhlet apparatus, and crude protein was calculated with the total nitrogen multiplied by 6.25. Glycogen content was detected by anthrone‐sulfuric acid colorimetry according to Chen, Yang, and Gu (2005), and polysaccharide content was measured by the phenol‐sulfuric acid method using glucose as the standard (Dubois, Gilles, Hamilton, Rebers, & Smith, 1956). All chromatographic assays were conducted with the phenol‐sulfuric acid method.

### 2.3 Extraction and isolation

The homogenates of oyster (*C. rivularis*, 200 g) were extracted using three methods: water, alkaline, and enzyme. The methods were performed as follows:

Water method: added 500 ml of water to the oyster homogenate and incubated at 80°C water for 2 hr and then cooled and centrifuged at 5,500 *g* for 20 min. The upper solution was collected for polysaccharide analysis (Gao, Zhao, Wang, & Luan, 2014); alkaline method: added 100 ml of 30% (w/v) potassium hydroxide (KOH) solution to the oyster homogenate and incubated at 100°C in water for 1 hr, cooled, and centrifuged. The polysaccharide content of the upper solution was analyzed at neutral pH (Chen, Yang, & Gu, 2005; Song et al., 2011); enzyme method: added 2 g of pepsin powder (60,000 U/g enzyme activity) to oyster homogenate and incubated at 50°C, pH 3.0 for 4 hr, inactivated the enzyme at 100°C for 10 min and centrifuged, and detected the polysaccharide content at neutral pH (Zhu et al., 2008). The polysaccharide extraction efficiency was defined as follows: ERP (%) = C_1_/C_0_ × 100%, where ERP is the extraction rate of the polysaccharide, C_1_ is the concentration of polysaccharide in the extracted solution, and C_0_ is the concentration of polysaccharide (wet weight) in the homogenate.

The alkaline extraction was selected as the best method according to the extraction rate of the polysaccharide. The extraction process was performed at different KOH concentrations (0%, 10%, 20%, 30%, and 40%), at increasing incubating temperatures (50, 60, 70, 80, and 90°C), and at increasing duration of time (30, 60, 90, 120, and 150 min). On the basis of a single‐factor test for polysaccharide, an orthogonal test was set up according to the L9 (3^4^) orthogonal table. The ERP was used as an indicator. Meanwhile, KOH concentration, extraction temperature, and time were considered as three factors. Each factor was tested in triplicate. The extract was filtered through a Whatman Nr1 filter paper, and the filtrate was then concentrated with a rotary evaporator at 60°C under reduced pressure. The proteins in the extract were removed using Sevag reagent, and after removal of the Sevag reagent, the concentrate was mixed with three times its volume of 95% ethanol then the mixture was maintained overnight at 4°C to precipitate the polysaccharides. The precipitate was collected after centrifugation at 3,000 r/min for 10 min and then dialyzed with moving water for 48 hr to obtain crude polysaccharides by lyophilization.

To remove fine particles, DEAE‐52 cellulose was first soaked in water until it was fully expanded. The expanded DEAE‐52 cellulose was then soaked in sodium hydroxide (NaOH, 0.5 M) solution for 30 min and washed to neutral pH by distilled water. The mixture was soaked in HCl (0.5 M) for 30 min, washed to neutral pH by distilled water, and then soaked in NaOH (0.5 M) again. As a result, the DEAE‐cellulose that was treated with 0.5 M of sodium chloride (NaCl) for 12 hr was transformed to Cl‐type. A column (2.6 × 80 cm) was filled with DEAE‐cellulose to the required density (without air bubbles) and the 2 to 3 cm gap at the top of the column was covered with water. After the preprocessing of DEAE‐cellulose in the column, the sample was douched using distilled water at concentrations of 0, 0.1, 0.2, 0.3, 0.4, 0.5 M of NaCl. The eluent flow rate was controlled at 48 ml/hr and collected automatically with 5 ml in each test tube, and then, each sample was characterized by its absorption peak. The sulfuric acid‐phenol method was used as a supplementary detection method. The fractions with major polysaccharide content were combined and further purified by a Sepharose 2B gel column (1.6 cm × 100 cm) equilibrated with 0.2 M phospharate (pH 6.8). The eluent was 0.2 M phospharate (pH 6.8), and the flow rate was 12 ml/hr. The major fraction was then collected, concentrated, desalted by dialysis, and then vacuum freeze‐dried (EYELA, FDU1110 Tokyo) to obtain the homogeneous polysaccharide.

### 2.4 Structural characterization of oyster polysaccharide

#### 2.4.1 Homogeneity analysis and molecular weight determination

The homogeneity and molecular weight (MW) of the polysaccharide samples were determined by high‐performance gel permeation chromatography (LC‐20AT), with a Sugar KS‐804 column (7.8 mm × 300 mm, TOSOH, Japan) and a differential refractive index detector (RID‐10A). For each run, 20 μl of sample solution (2 mg/ml) was injected into the liquid chromatography system and eluted with 0.1 M of NaNO_3_ with a flow rate of 1 ml/min. The column was calibrated with standard Dextrans (seven different weights: 10, 20, 43.5, 68. 5, 105, 500, and 2,000 kDa). The calibration curve of the log (MW) versus elution time (*t*) is: Log MW = −0.82105*t* + 11.5057 (*R* ^2^ = 0.9973).

#### 2.4.2 Monosaccharide compositional analysis

The monosaccharide composition of polysaccharides was evaluated through gas chromatography. Ten milligrams of the polysaccharide was dissolved in 4 ml 2 M trifluoroacetic acid and hydrolyzed at 100°C for 4 hr. After the reaction, the remanent TFA was removed by co‐evaporation at reduced pressure with ethyl alcohol added. The subsequent treatment of the resultant dry hydrolysate with acetic anhydride and pyridine afforded the corresponding alditol acetate which was analyzed by gas chromatography (GC7900) fitted with a flame ionization detector and TM‐5 column. The analytical conditions were 3 min at 180°C; from 180 to 230°C at 10°C/min and keeping for 20 min at 230°C; from 230 to 240°C at 5°C/min and keeping for 20 min at 240°C; and from 240 to 250°C at 5°C/min and keeping for 30 min at 250°C (Zhu et al., 2008).

#### 2.4.3 Methylation analysis

The purified fraction was methylated according to the method of Needs and Selvendran (1993). The disappearance of the hydroxyl absorption peak in IR spectrum at 3,400 cm^−1^ indicated the methylation completion. The methylated products were depolymerized with 85% formic acid for 4 hr and then hydrolyzed with 2 M TFA for 6 hr at 100°C. The hydrolysate was then reduced and acetylated. The products were quantitatively analyzed by the GC‐MS method described by Yang (2013), and the products of methylated alditol acetates and their molar ratios were obtained.

#### 2.4.4 FT‐IR analysis

The purified fractions were identified using an FT‐IR spectrophotometer (FTIR‐850, Germany). The polysaccharides were pressed into KBr powder and then pressed into a disk. The spectrum was recorded within 4,000 to 400 cm^−1^.

#### 2.4.5 Nuclear magnetic resonance (NMR) spectroscopic analysis

The polysaccharide samples (30 mg) were ultrasonically dissolved (30 min) in D_2_O; then, they were subjected to NMR analysis (Bruker AV‐500). The spectrometer was operated at 500.13 MHz (^1^H) and 125.75 MHz (^13^C) according to the method described by Zhu et al. (2010, 2011).

## 3 RESULTS AND DISCUSSION

### 3.1 Chemical composition of *C. rivularis*

The general chemical composition of *C. rivularis* was analyzed. The whole viscera of *C. rivularis* contained 10.58 g/100 g of crude protein by wet weight and 1.97 g/100 g of fat by wet weight. The glycogen content in *C. rivularis* was 4.92 g/100 g wet weight, which is less than the glycogen content in *C. gigas* (7.05 g/100 g wet weight, Chen et al., 2005). Linehan, O'Connor, and Burnell (1999) observed fluctuations in glycogen content in Irish oysters (*C. gigas*), which started to synthesize and store glycogen after spawning. Lira et al. (2013) also indicated that the carbohydrates content of mangrove oysters in summer was higher than that in winter. The glycogen content in *C. rivularis* may have the same seasonal change trends. In this study, the selected material came from the aquatic products market, and *C. rivularis* did not in the spawning season; this may have caused the glycogen content to be less than *C. gigas*.

### 3.2 Extraction and purification of polysaccharide from *C. rivularis*

Water, alkaline solution, and enzyme are often used to extract the polysaccharide, so these three extraction methods were compared in the crude polysaccharide extraction from *C. rivularis*. The results indicated that the KOH solution had the highest extraction rate (90.14%), which means KOH can destroy the protein moiety associated with glycogen and increase the breaking of the N‐ and O‐connected bonds.

**Figure 1:**
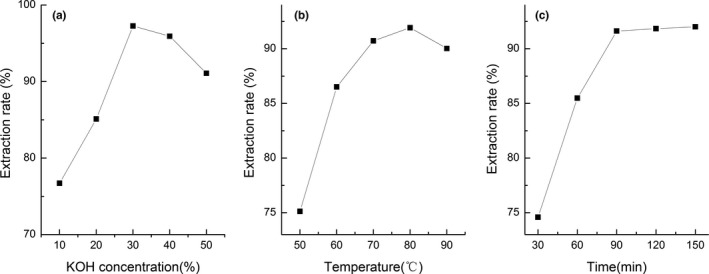
Relationship between KOH concentration (a), extraction temperature (b), and time (c) and extraction rate of glycogen from *Crassostrea rivularis*

The polysaccharide extraction rate was influenced by various factors (Figure 1). Figure 1a shows the effect of KOH concentration on the extraction rate. As shown in Figure 1a, the extraction rate increased rapidly when the KOH concentration ranged from 10% to 30% and then decreased. With the increasing KOH concentration, more N‐ and O‐type carbohydrate‐peptide bonds were destroyed which caused more glycogen to be released. But as the KOH concentration increased above 30%, the glycogen structure was destroyed. Sugar had an enolization reaction while C4 and C5 had a dehydration reaction, subsequently causing an unsaturated sugar alcohol group to be formed. The extracted solution was so sticky that it was difficult to filter which caused the extraction rate of glycogen to be lower. As shown in Figure 1b, the ERP increased when the temperature ranged from 50 to 80°C and then decreased between 80 and 90°C. High temperature led to protein denaturation and decreased the extraction rate.

Extraction time is another affecting factor in the polysaccharide's extraction for the time requirement of the exposure of the glycogen to KOH solution (Ye & Jiang, 2011). It was found that the ERP increased as extraction time ranged from 30 to 90 min and then increased slowly after 90 min (Figure 1c). Prolonged extraction time induced the degradation of polysaccharides and reducing of ERP. So, the optimum extraction time was 90 min.

The results of orthogonal design and variance analysis showed that the factor most influencing the extraction rate was extraction temperature, followed by KOH concentration, and extraction time (Table 1). In summary, the optimal extraction conditions were KOH concentration of 30% (w/v), temperature of 90°C and time of 120 min; under these conditions, the polysaccharide extraction rate was 92.36 ± 0.13%.

**Table 1:** Results of orthogonal test of glycogen from *Crassostrea rivularis* using the KOH extraction method

No.	Factors			ERP (%)
	KOH concentration (%)	Temperature (°C)	Time (min)	
1	1 (25)	1 (70)	1 (60)	83.32
2	1	2 (80)	2 (90)	85.56
3	1	3 (90)	3 (120)	89.92
4	2 (30)	1	2	88.30
5	2	2	3	92.27
6	2	3	1	90.58
7	3 (35)	1	3	85.76
8	3	2	1	87.47
9	3	3	2	89.45
K_1_	258.80	257.38	261.37	
K_2_	271.15	265.30	263.31	
K_3_	262.68	269.95	267.95	
k_1_	86.27	85.79	87.12	
k_2_	90.38	88.43	87.77	
k_3_	87.56	89.98	89.32	
R	4.11	4.19	2.20	

The Sevag reagent was used to remove the protein in the extracted solution. The protein removal rate was increased with repetition and achieved the highest rate of 91.6% at five times. There was no absorption peak at 280 nm after ultraviolet scanning. The crude polysaccharide (CG) contained 80.72% total sugar, 1.96% protein, and 0.16% fat.

**Figure 2:**
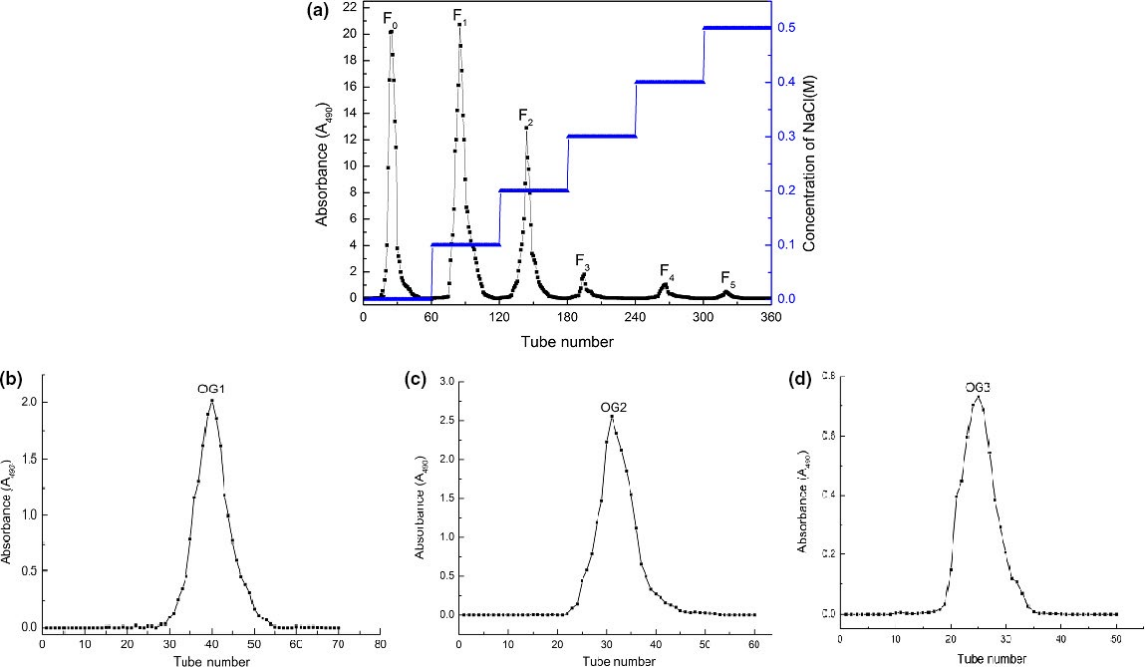
Elution profiles of crude polysaccharide by ion exchange chromatography on a column of DEAE‐52 cellulose (a) and Elusion profiles of F0, F1, F2, respectively, by gel filtration chromatography on a column of Sepharose 2B, respectively (b, c, d)

The crude polysaccharide was first separated through an anion‐exchange column of DEAE‐52 cellulose. The column was eluted with different NaCl solution (0, 0.1, 0.2, 0.3, 0.4, and 0.5 M), and six independent elution peaks were obtained (F_0_, F_1_, F_2_, F_3_, F_4_, F_5_; Figure 2a). The F_0_, F_1_, and F_2_ fractions were collected, concentrated, dialyzed, reconcentrated, and loaded onto a column of Sepharose 2B. The column was eluted with 0.2 M phospharate (pH 6.8), and the resulting elution was collected. Figure 2b–d show each fraction generated one single elution peak affording OG1, OG2, and OG3, respectively.

### 3.3 Structure characteristics of polysaccharide from *C. rivularis*

**Figure 3:**
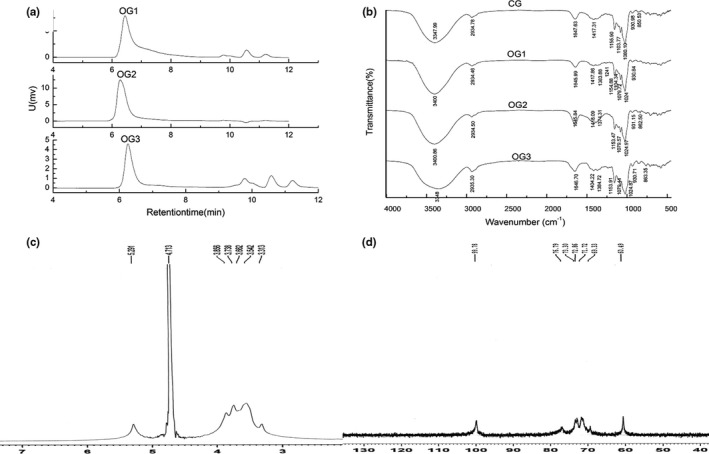
(a) HPGPC elution profiles of OG1, OG2, and OG3; (b) The FTIR spectra of CG, OG1, OG2, and OG3 from *Crassostrea rivularis*; (c) 1H‐NMR spectrum of OG2; (d) 13C‐NMR spectrum of OG2. HPGPC, high‐performance gel permeation chromatography

High‐performance gel permeation chromatography profile (Figure 3a) showed OG1, OG2, and OG3 had one main and symmetrically sharp peak, indicating that the three fractions were homogeneous polysaccharides. The MW of OG1, OG2, and OG3 were 1,660, 2,270, and 2,330 kD, respectively. Table 2 shows the chemical compositions of OG1, OG2, and OG3. The three fractions were polysaccharides with protein content less than 1.5%, which means the Sevag method was efficient in removing protein.

**Table 2:** Chemical compositions of three fractions of polysaccharides from *Crassostrea rivularis*

Sample	Molecular weight (Da)	Total sugar (%)	Protein (%)	Monosaccharide composition (mol %)						
				Rhamnose	Mannose	Ara	Fucose	Xylose	Glucose	Galactose
OG1	1.66 × 10^6^	94.45	0.61	90.05	8.71	—a	—	—	—	—
OG2	2.27 × 10^6^	97.82	0.55	—	—	—	—	—	98.23	—
OG3	2.33 × 10^6^	86.68	1.24	57.47	—	—	4.06	12.11	—	22.56

Note

a Not detectable; Ara: arabinose.

OG1 and OG3 were composed mainly of rhamnose. OG1 was composed mainly of rhamnose (90.05%) and a small amount of mannose (8.71%). In OG3, the ratio of Rha:Gal:Xyl:Fuc is 14:5.5:3:1. Yin, Zheng, Zheng, and Wang (2009) isolated a kind of acidic polysaccharide from *Spirulina platensis* which was composed of D‐rhamnose, D‐xylose, D‐glucose, and D‐mannuronic acid with a molar ratio of 65.5:19.5:9:6. In this research study, OG1 and OG3 had similar major composition, which were rhamnosan. According the results of the monosaccharide composition, OG1 and OG3 may be same as the acid polysaccharide from *S. platensis. S. platensis* was ingested by oysters and may be accumulated in their bodies. OG2 was composed only of glucose (98.23%), which indicated it was glycogen.

The FT‐IR spectrum of CG, OG1, OG2, and OG3 is shown in Figure 3b. The strong and wide absorption band of approximately 3,200 to 3,600 cm^−1^, 2,800 to 3,000 cm^−1^, and 1,640 to 1,650 cm^−1^ corresponded to O–H, C–H, and C–O stretching vibrations, respectively. The bands in the approximate region of 3,400 cm^−1^, 2,934 cm^−1^, 1,645 cm^−1^, and 1,374 cm^−1^ in OG2 were characteristic absorptions of polysaccharides. Peaks of 1,153 cm^−1^, 1,079 cm^−1^, and 1,024 cm^−1^ were the special absorption of pyranoside. Peaks of approximately 900 cm^−1^ to 1,200 cm^−1^ were due to C–O–C stretching vibrations of the sugar ring and C–O bending vibrations. The 862 cm^−1^ absorption was due to α‐glycosidic bonds.

To elucidate the structure of OG2, NMR spectra (Figure 3c and d) were determined. Based on the analysis of the chemical composition of OG2, it was shown that OG2 was a glucan. According the ^1^H‐NMR spectrum, the chemical shifts at 5.29 ppm were the anomeric hydrogens of the α‐glucopyranose residues.

The intensive peaks in the ^1^H‐ and ^13^C‐NMR spectra of OG2 were associated with the 1,4‐D‐Glcp residue. The resonances of δ99.78 ppm (Figure 3d) were attributed to the anomeric carbon atoms, indicating anomeric configuration for all monosaccharide residues in OG2. There are no carboxyl, acetyl, and amido groups in OG2 because no chemical shift was observed in the region above δ170 ppm. It was concluded that all sugars were pyran rings as the C‐1 resonance bands of the furan ring at δ107 to δ109 ppm were not observed. The carbon peak at δ76.79 ppm was indeed C‐4 of the 1,4‐D‐Glcp residue, and it was shifted about 7 ppm downfield compared with the resonance of standard D‐Glcp (Agrawal 1992). The other intensive signals at δ72.86, 73.3, 71.72, and 60.49 ppm represented C‐2, C‐3, C‐5, and C‐6 of the 1,4‐d‐Glcp residue, respectively (de Bruin, Parolis, & Parolis, 1992). The spectroscopic signals for 1,4,6‐D‐Glcp and 1‐D‐Glcp residues were less intensive and some could hardly be observed due to their small amount in OG2. Therefore, these observations further confirmed that OG2 was a glycogen, consistent with early reports by Yang (2013) and Matsui, Kakut, and Misaki (1996).

**Figure 4:**
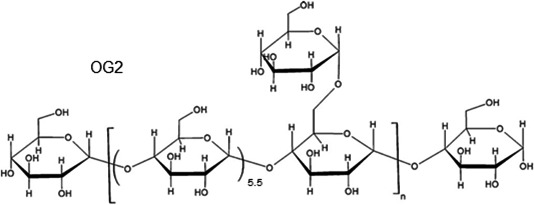
Structure of the OG2

The fully methylated products were hydrolyzed with acid, converted into alditol acetates, and analyzed by GC‐MS. Experimental results showed that OG2 consisted solely of glucose and appeared to have an overwhelming percentage of 1,4,5‐tri‐O‐acetyl‐2,3,6‐tri‐O‐methyl‐D‐glucitol with some 1,4,5,6‐tetra‐O‐acetyl‐2,3‐di‐O‐methyl‐D‐glucitol (Table 3). The glycogen nature of the OPS was confirmed because of its typical glycogen linkage characteristics. The ratio of 1,4‐linked‐glucose to 1,4,6‐linked‐glucose was 6.5, suggesting that the oyster glycogen chain branched out every 6.5 glucose residues on average. In addition, 2,3,4,6‐Me_4_‐Glc were also detected, suggesting the existence of a trace amount of 1‐linked glucose (Figure 4).

**Table 3:** GC‐MS data of OG2 methylated sugar residues

Methylated sugar residue	Retention time (min)	Main fragments (m/z)	Type	Mol (%)
2,3,4,6‐Me_4_‐Glc	14.861	71.87.101.117.129.145.162.205	Glc‐(1→	1.1
2,3,6‐Me_3_‐Glc	17.061	87.99.101.113.117.233	→4)‐Glc‐(1→	80.7
2,3‐Me_2_‐Glc	19.109	101.117.129.142.201.231	→4,6)‐Glc‐(1→	12.4

Yang et al. (2013) researched the structure of the glycogen from the oyster (*Ostrea talienwhanensis Cross)* and found that the glycogen has the structure of α‐(1→4) D‐linked glucose which has the branch every 6.6 residues on average; and trace amounts of 1,2,4‐linked, 1,6‐linked, and 1,3‐linked glucoses were found, which were different from the composition of glycogen from *C. rivularis*.

Miller, Dodd, Ormrod, and Geddes (1993) researched the glycogen extracted from *Perna canaliculus* (NZ green‐lipped mussel), which has anti‐inflammatory activity. However, this activity was lost if the glycogen extract was treated with KOH or proteinase K, suggesting that the anti‐inflammatory properties resided within a protein moiety associated with glycogen. In this research, OG2 is a kind of glycogen isolated from *C. rivularis* by KOH extraction and may be used as an ideal model for the investigation of polysaccharide structure–activity correlation because of its simple composition and linkage structure.

## 4 CONCLUSIONS

Oyster (*C. rivularis*) polysaccharide was extracted using KOH solution. The optimal extraction conditions were KOH concentration of 30% (w/v), temperature of 90°C, and time of 120 min; under these conditions, and the polysaccharide extraction rate was 92.36 ± 0.13%. Crude polysaccharide was later purified by DEAE‐52 cellulose and Sepharose 2B gel. Three fractions, OG1, OG2, and OG3, were identified with a weight‐average MW of 1,660, 2,270, and 2,330 kD, respectively. OG1 and OG3 were rhamnosan, which were similar to the acid polysaccharide from *S. platensis*, and OG2 was glycogen with α‐(1→4)‐D‐linked glucose. The branch chain existed in every 6.5 glucose residues on average, which is →4,6)‐α‐D‐Glc‐(1→ and a trace amount of α‐D‐Glc‐(1→ branched units. Further bioactivities evaluation of this special structure polysaccharide from *C. rivularis* will be important for their application in food fields.

## CONFLICTS OF INTEREST

The authors declare no conflict of interest.
